# Two cases of brainstem neurocysticercosis removal: operative video

**DOI:** 10.3171/2019.10.FocusVid.19458

**Published:** 2019-10-01

**Authors:** Xiaochun Zhao, Robert T. Wicks, Evgenii Belykh, Colin J. Przybylowski, Mohamed A. Labib, Peter Nakaji

**Affiliations:** 1 Department of Neurosurgery, Barrow Neurological Institute, St. Joseph’s Hospital and Medical Center, Phoenix, Arizona; and; 2 Irkutsk State Medical University, Irkutsk, Russia

**Keywords:** brainstem, cyst, far lateral approach, inflammation, larva, neurocysticercosis, subtemporal approach, tapeworm, video

## Abstract

Neurocysticercosis is primarily managed with anthelminthic, antiepileptic, and corticosteroid therapies. Surgical removal of the larval cyst is indicated when associated mass effect causes neurological symptoms, as demonstrated in two cases. Cyst resection was achieved via the far lateral approach for a cervicomedullary cyst in one patient and via the subtemporal approach for a mesencephalic cyst in another. The cyst wall should be kept intact, when possible, to avoid dissemination of the inflammation-evoking contents. As the contents are usually semisolid and can be removed via suction, it is not necessary to remove the gliotic capsule or adherent portions of the cyst wall in highly eloquent locations.

The video can be found here: https://youtu.be/GqbaJu5sy1o.

## Transcript

**0:20** Two cases of neurocysticercosis of the brainstem treated with microsurgical resection by the far lateral and subtemporal approaches.

**0:30** Case 1 is a 36-year-old female who presented with headache, rapidly progressive dysphagia, and left-sided numbness. MRI scan of the brain disclosed an enhancing mass presenting towards the right side of the brainstem, which is seen here at the cervicomedullary junction. At this point, it was not clear what the diagnosis was, but the patient was progressing rapidly, and surgery was felt to be indicated.

**0:57** The patient was placed into the park-bench position with the left side down, and a right-side reverse hockey-stick incision was planned for a far lateral approach. This approach will allow us to approach the brainstem from underneath, anterior and below the lower cranial nerves. The surgical strategy here is to make as direct an approach as possible to the brainstem without retracting on the brainstem or on the cranial nerves. For this, because the lesion presents laterally and anteriorly, we need a very extensive far lateral craniotomy including upper condylectomy. We are looking down upon the surgical site.

**1:40** We are drilling now the upper part of the right condyle. The upper part of the head is to the left and the lower part to the right. The upper portion of the condyle, above its junction with C1, is drilled away until is relatively flat so the dura can be reflected over this area. We see the lower brainstem and the cerebellum, as well as the eleventh nerve. The arachnoid is open sharply, which reveals the dentate ligament. The dentate ligament is sectioned. Opening a corridor towards the anterior brainstem, we can quickly come down on to the brainstem where a bulge is noted anterior to the twelfth cranial nerve.

**2:33** A very small opening is made when it becomes apparent what this is. The characteristic appearance of neurocysticercosis is immediately clear to the surgeon, and therefore, a very conservative process of gradually bringing the slippery cyst contents out without disturbing the wall is carried out. This has a very favorable prognosis, and so it is even more essential to remove it without disturbing the delicate neurovascular structures in the area or the brainstem any more than necessary.

**3:08** In this case, the patient made an excellent recovery, there was no residual on MRI, and the symptoms disappeared over a period of weeks. Here we can see the extent of the condylectomy, which does include the medial and superior portion of the condyle without disturbing the articular surfaces between the occiput and C1. The patient did not develop any instability over the ensuing years. Pathology confirmed neurocysticercosis. The patient went on to further treatment with albendazole. A more comprehensive review of systems did not reveal a reason for her to have contracted this condition.

**3:46** Case 2 is a 39-year-old male who presented with left-sided weakness and clumsiness. Imaging shows a hypodense lesion on CT scan in the right midbrain which comes into the ambient cistern. The same lesion is seen on MRI scan, and it is notable that this lesion is cystic with a great deal of mass effect but does not enhance.

**4:10** We see a small nodule located laterally on the surface which did appear to enhance while the center appeared to be clear. At this point, the patient was observed, but over a period of time, the lesion actually expanded and caused progressive symptoms and mass effect on the brain. We did suspect neurocysticercosis at this time, but because of the patient’s progressive left hemiparesis, loss of pain and temperature sensation, and right-sided clumsiness, felt that he was a candidate for surgical treatment.

**4:39** The patient was treated with a subtemporal approach, positioned with the head turned over to the left side to reveal the right subtemporal area, which was then tipped down to allow the temporal lobe to fall away from the middle fossa floor. A straight or curvilinear incision can be used in order to fashion a rectangular craniotomy whose floor is completely flat to the middle fossa. The anatomy of the subtemporal approach is depicted here. In this case, the top of the petrous bone has been drilled away and the tentorium cut, which reveals the access to the midbrain, pons, and upper cerebellum.

**5:23** The surgical strategy here includes making an approach underneath the temporal lobe and sliding up over the surface of the tentorium until we come to the lateral midbrain. At this point, the tentorium can be opened, taking great care to preserve the fourth cranial nerve, or if the lesion presents to the subarachnoid space, the lesion can simply be opened. The strategy for neurocysticercosis is somewhat different than for other lesions because a great deal of exposure is not required. Dissection of the cyst contents is usually quite simple.

**5:53** Here we see the dural opening with the middle fossa floor uppermost in the field and the superior part inferiormost. A simple subtemporal dissection directly down takes us almost immediately onto the surface of the midbrain laterally, where the lesion is identified. Even the process of respiration causes it to begin to herniate out once this capsule has been opened. The materials are usually quite slick and mobile and are easily delivered from within the center part of the lesion. It is not necessary to remove the wall beyond this. At this point, the worm is usually not alive any longer. It is actually the process of degeneration that sometimes causes them to swell and cause symptoms. Gradual traction allows it to be delivered from the midbrain. Great care should be taken not to provide traction, and if any resistance is felt, further dissection should be carried out. Other cyst debris are delivered out through the same opening, taking care not to disturb the wall of the brainstem any more than necessary. Here we see into the interior of the cavity.

**7:10** Pathology revealed this again to be neurocysticercosis with the characteristic pathologic finding of the scolex. The gross cyst seen in the picture on the lower right. Postoperative imaging revealed the empty cavity and relative relaxation of the brainstem. The patient’s symptoms improved, and gradually the anatomy returned to normal.

**7:34** Pearls and pitfalls. In these cases, it is advantageous to try to preserve the cyst wall as long as possible and to evacuate the contents with suction rather than allowing them to spill. It is important not to pull on the surrounding structures, which are usually not attached tightly to the cyst and are usually quite eloquent in this location. Patients should be treated with antiparasitic therapy if feasible but, if not, microsurgical removal offers an excellent way to reduce mass effect and provides prompt resolution of the patient’s symptoms.
